# von Willebrand factor-binding protein (vWbp)-activated factor XIII and transglutaminase 2 (TG2) promote cross-linking between FnBPA from *Staphylococcus aureus* and fibrinogen

**DOI:** 10.1038/s41598-023-38972-3

**Published:** 2023-07-19

**Authors:** Chiara Motta, Angelica Pellegrini, Stefano Camaione, Joan Geoghegan, Pietro Speziale, Giulia Barbieri, Giampiero Pietrocola

**Affiliations:** 1 Department of Molecular Medicine, University of Pavia, Pavia, Italy; 2 Department of Biology and Biotechnology “Lazzaro Spallanzani”, University of Pavia, Pavia, Italy; 3 Institute of Microbiology and Infection, University of Birmingham, Edgbaston, Birmingham, B15 2TT UK

**Keywords:** Biochemistry, Biological techniques, Microbiology, Molecular biology

## Abstract

The secreted von Willebrand factor-binding protein (vWbp) from *Staphylococcus aureus* interacts with the coagulation factors prothrombin and fibrinogen (Fbg), leading to the non-proteolytic transglutaminase activation of Factor XIII (FXIII). In this study we found that vWbp-activated FXIII catalyses the incorporation of amino-donor dansylcadaverine into region A of fibronectin-binding protein A (FnBPA). Incubation of Fbg with recombinant region A of *S. aureus* Fbg-binding proteins FnBPA, FnBPB, ClfA or ClfB in presence of vWbp-activated FXIII resulted in the formation of high molecular heteropolymers with FnBPA only, suggesting a specificity of the cross-linking reaction between fibrin(ogen) and the staphylococcal surface. As previously observed, cross-linking sites were mapped to the α-chain and the N1 subdomain of fibrin(ogen) and region A of FnBPA, respectively. Comparable results were obtained when tissue tranglutaminase-2 (TG2) was tested for cross-linking of FnBPA and Fbg. Of note, FnBPA-mediated covalent cross-linking promoted by vWbp-activated FXIII was also observed when bacteria were allowed to attach to fibrin(ogen). Together these findings suggest a novel pathogenetic mechanism by which the transglutaminase action of FXIII and/or TG2 contributes to entrapment and persistence of *S. aureus* in blood and host tissues.

## Introduction

*Staphylococcus aureus* is a human pathogen well-known for its ability to cause community- and health care-associated infections ranging from mild skin infections to severe diseases including pneumonia, sepsis and toxic shock syndrome^1^. Moreover, this bacterium has been identified as the major etiological agent of infective endocarditis, having a propensity to adhere to fibrin-platelet clots on damaged cardiac valves, referred to as vegetations^2,3^. In addition to becoming increasingly resistant to antibiotics, *S. aureus* is a master in adapting to its host by avoiding almost every facet of the immune system^4^. The high pathogenetic potential of *S. aureus* is due to the expression of a plethora of virulence factors, most of which are surface proteins covalently anchored to the wall peptidoglycan (CWA, cell wall anchored proteins). These proteins, called MSCRAMMs (Microbial Surface Component Recognizing Adhesive Matrix Molecules), specifically recognize host matrix components^5^. The fibronectin (Fn)-binding MSCRAMMs FnBPA and FnBPB (Fn-binding protein A and B) are two structural and functional homologous CWA proteins, among the most intensively studied adhesins of *S. aureus*. FnBPs have been found to bind Fn^6^, fibrinogen (Fbg)^7,8^, plasminogen^9^, elastin^10^ and histones^11^. Furthermore, FnBPs play an important role in the formation of biofilm^12^ and are involved in the invasion of a variety of non-phagocytic cell lines^13–17^. Structurally, FnBPs comprise two distinct domains, the N-terminal and the C-terminal, which are similarly organized in the two proteins. The N-terminal domain comprises the region A, organized in three separately folded subdomains N1, N2 and N3^18^. N2 and N3 together form IgG-like folds typical of MSCRAMM proteins, with a potential to bind ligands such as Fbg by the “dock, lock and latch” (DLL) mechanism^19,20^. The C-terminal domain comprises 10–11 tandemly repeated Fn binding units^18^. In addition to CWA proteins, *S. aureus* expresses a multitude of secreted proteins/peptides that compromise innate immune response^21^ and two hemostasis factors, the coagulase (Coa)^22^ and the von Willebrand factor-binding protein (vWbp)^14,23,24^. Both Coa and vWbp are able to independently bind and non-proteolytically activate prothrombin (ProT) to thrombin, which in turn cleaves Fbg to fibrin and forms fibrin cables^25–27^. By effect of this action, both coagulases contribute to the pathogenicity of the microorganism as proved in several animal models^26,28^. Coa and vWbp have significant structural and functional similarities: in fact, the N-terminal regions of vWbp and Coa are structurally similar, and able to interact with and activate ProT in an analogous mechanism^29^. Moreover, both the N-and C-terminal moieties of vWbp and Coa bind Fbg, although with different affinities^30^. Differently from Coa, vWbp interacts with vWF and Fn^27^. vWbp-activated ProT in the presence of Fbg triggers the non-proteolytic activation of plasma circulating Factor XIII (FXIII) in vitro^27^. Activated-FXIII catalyses post-translational protein-modification reactions involving the cross-linking of lysine and glutamine residues to produce ε-(γ-glutamyl)-lysine isopeptide bonds (transglutaminase activity) and formation of γ-chain dimers and α-chain multimers in fibrin^27^. This reaction provides stability and resistance to degradation by plasmin of fibrin cables in clots and abscess communities.

In physiological conditions FXIII is directly activated by thrombin and can induce the formation of isopeptide bonds that ligate adjacent fibrin monomers in the clot^31–33^. Thrombin-activated FXIII also introduces cross-links between FnBPA and Fbg or Fn^34^.

Apart from FXIII, transglutaminase activity is associated to a number of transglutaminase isoforms, namely TG1, TG2, TG3, TG4, TG5, TG6, TG7^35^. TG2 (tissue transglutaminase), the most studied isoform, is found in fibroblasts, vascular endothelium, smooth muscle cells, and in the extracellular matrix (ECM) of various tissues and of the arterial walls^35^. Many intra- and extracellular proteins have been identified as TG2 substrates for TG2-mediated cross-linking reactions. In the ECM, TG2 initiates cross-linking of structural proteins such as collagen, Fn, Fbg and laminin, leading to the formation of an ECM scaffold that displays an increased stability and rigidity^36–38^.

In this study, we analysed vWbp expression during *S. aureus* growth and the localization of the protein after its secretion. Furthermore, we investigated whether vWbp-activated FXIII and TG2 are able to form cross-links between Fbg and the staphylococcal adhesin FnBPA and demonstrated that the covalent cross-linking reaction is effective when *S. aureus* cells attach to Fbg substrate.

## Results

### vWbp is predominantly expressed in the exponential growth phase and recognized by IgG in serum from patients with *S. aureus* infective endocarditis

**Figure 1:**
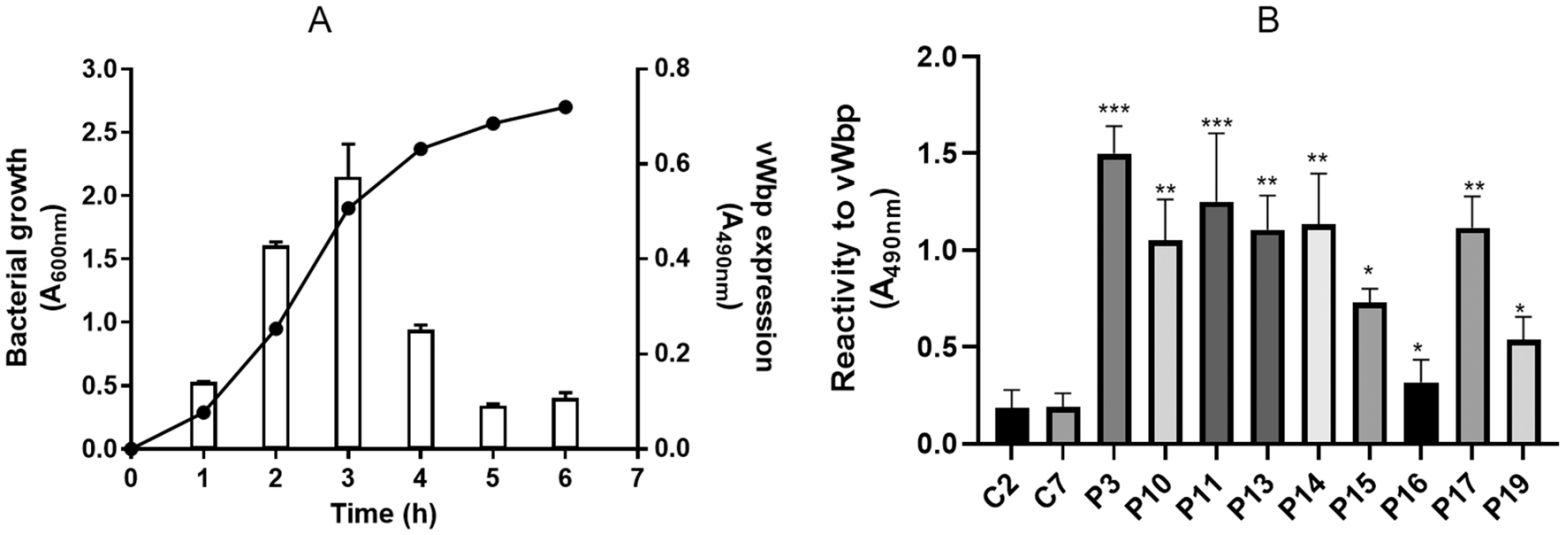
Expression of vWbp during bacterial growth and protein detection in sera from patients with infective endocarditis. (**A**) At the indicated times, the bacterial culture supernatants were collected, filtered and then immobilized onto microtiter wells. The presence of vWbp in the wells was revealed using anti-vWbp IgG followed by secondary HRP-conjugated IgG (right axis). Bacterial growth curve is also reported (left axis). (**B**) To test the immunological reactivity against vWbp, microtiter wells coated with vWbp were probed with IgG isolated from sera o human patients affected by staphylococcal endocarditis (P). IgG from healthy donors were used as controls (C2, C7). Bound antibody was detected by the addition of secondary HRP-conjugated IgG to the wells. Statistically significant differences between patients and controls are indicated (*P < 0.05; **P < 0.01; ***P < 0.001). Data are expressed as means ± S.D. of tests performed in triplicate.

vWbp is a secreted protein^23,24^*.* To determine its optimal expression, a mutant LAC strain deficient in the protein A gene (*spa*) of *S. aureus* was grown in BHI during different growth phases. The supernatant was collected from the culture at regular time intervals, immobilized onto ELISA microtiter wells and the level of the protein detected using a polyclonal anti-vWbp antibody. As shown in Fig. 1A, vWbp is predominantly expressed in the exponential phase of growth (2–4 h) with a maximum expression at 3 h post-inoculum. The specificity of the ELISA assay was further confirmed by a Western immunoblotting, where a protein with a molecular mass corresponding to vWbp (60 kDa) was detected in the supernatants of *S. aureus* LAC *spa* (data not shown).vWbp is involved in the initiation of infective endocarditis in animal models^39^. Thus, to determine whether vWbp is an immunological active component and expressed in vivo we examined the IgG level against vWbp in previously characterized sera from patients with *S. aureus* infective endocarditis^40^. Although variability from one serum to another was noted, IgG from all patients exhibited a reactivity with vWbp significantly higher than that observed with IgG from healthy donors, indicating the in vivo expression of vWbp and its relevance as an antigen (Fig. 1B).

### Secreted vWbp rebinds to the cell surface and maintains its functional activity

**Figure 2:**
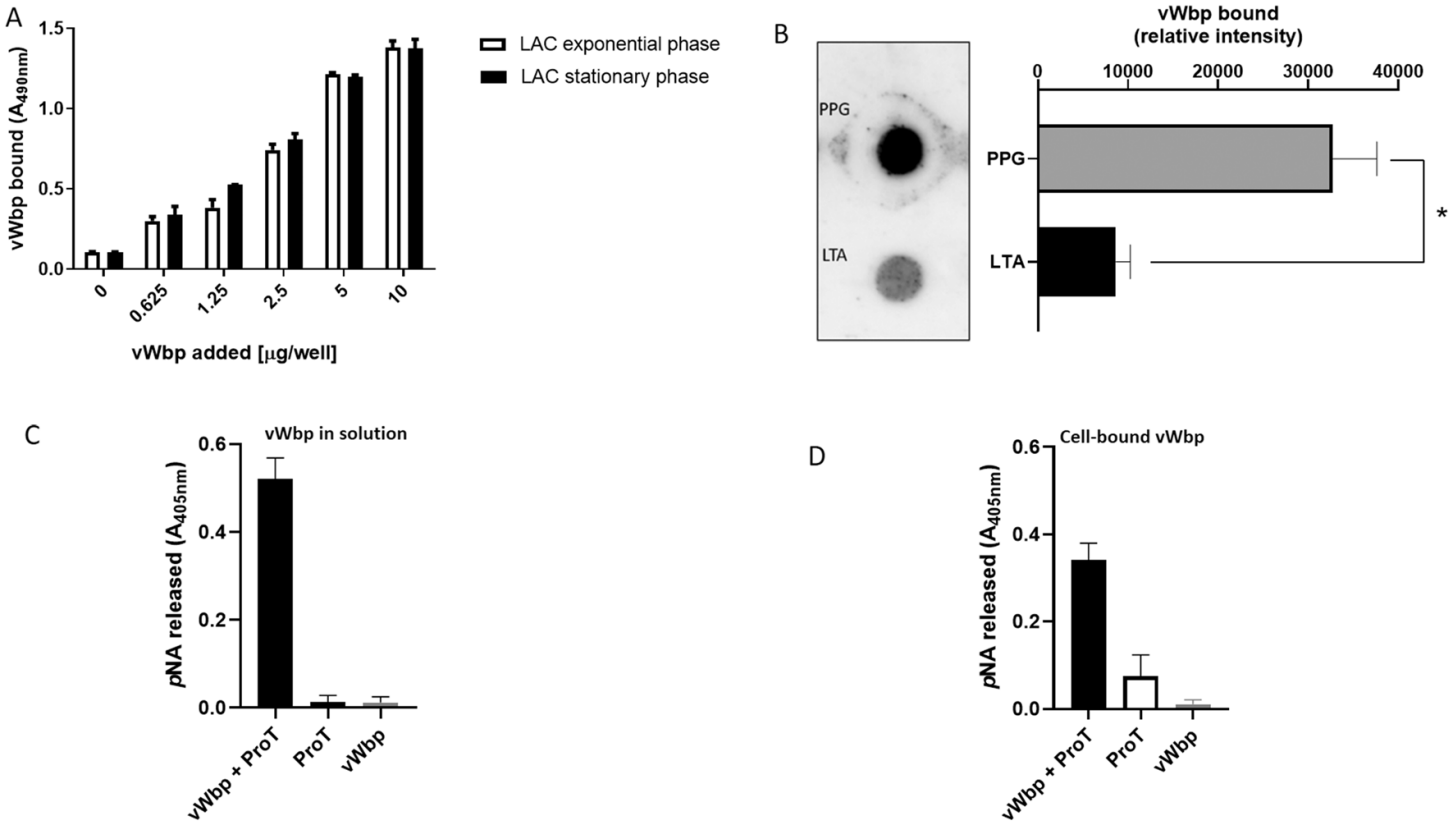
vWbp can associate to the bacterial cell surface via peptidoglycan and lipoteichoic acid and remains functionally active. (**A**) Binding of increasing concentrations of biotinilated vWbp to *S. aureus* LAC cells from exponential and stationary phases immobilized onto microtiter wells was determined by addition of avidin-peroxidase. (**B**) Binding of biotinilated vWbp to PPG or LTA dotted onto PVDF membrane was detected incubating the membrane with avidin-peroxidase. Densitometric analysis of vWbp bound to PPG or LTA is shown on the right. Statistically significant difference is reported (*P < 0.05). (**C**) and (**D**) ProT activation by soluble vWbp (**C**) or vWbp bound to immobilized *S. aureus* LAC cells (**D**) was detected by measuring pNa released from S2238 hydrolysis. Data are expressed as means ± S.D. of triplicate tests.

To investigate whether vWbp could be captured and retained on the bacteria surface, *S. aureus* LAC cells from both exponential and stationary phases were spotted onto ELISA microtiter wells, incubated with increasing concentrations of biotyn-labeled vWbp and association of vWbp to bacteria revealed with avidin-peroxidase. As shown in Fig. 2A, the protein bound to the surface of both exponentially and stationary phase cells, suggesting that the cell surface structures responsible of interaction with the protein are expressed in both stages of growth.

To identify these structures, we compared the rebinding of biotinylated vWbp to the surface of *S. aureus* LAC wt and its sortase A (*srtA*) deletion mutant. In these conditions, no significant difference was observed in binding of vWbp to bacterial surface, suggesting that vWbp binding is not mediated by CWA proteins tethered by sortase A (Fig. 1S). In support of this, none of a collection of sortase A-anchored proteins (ClfA-B, FnBPA-B, CNA, SdrC, SdrD, SdrE, IsdB, IsdH and SpA) displayed interaction with biotin labelled vWbp in an ELISA assays (data not shown).

It should be noted that *S. aureus* cell wall displays on the surface a variety of other molecules such as peptidoglycan (PPG), lipotheicoic acid (LTA), sortase B-anchored IsdC protein and a number of lipoproteins. To test the possible involvement of PPG and lipoteichoic acid (LTA)^41^ in vWbp binding, highly purified PPG and LTA from *S. aureus* were spotted onto a PVDF membrane, followed by incubation with biotinylated vWbp. Both LTA and PPG interacted with vWbp, with PPG showing a significantly higher binding than LTA (Fig. 2B). Due to the biochemical complexity of the staphylococcal surface we do not exclude that vWbp can interact with additional components of the cell wall.

Next, we investigated whether soluble and surface attached vWbp retain a comparable ability to activate ProT. As shown in Fig. 2C and D, both the forms of vWbp exhibited similar activating effect on ProT^42^, while a negligible activation of ProT was observed incubating the zymogen with substrate in the absence of vWbp. Likewise, no intrinsic enzymatic activity was observed incubating the substrate with vWbp alone.

### vWbp activated-FXIII elicits the formation of cross-links between FnBPA N1N2N3 domain and fibrinogen α chain

**Figure 3:**
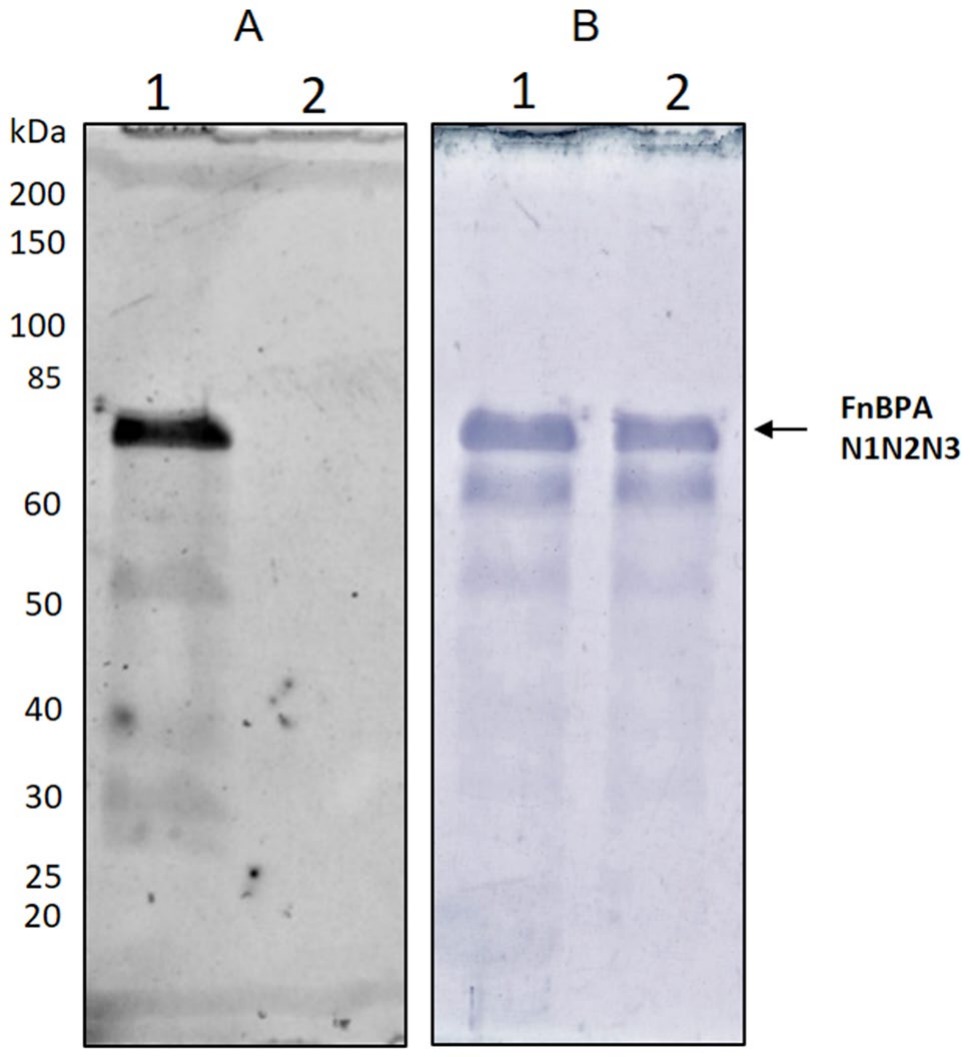
Incorporation of dansyl cadaverine into FnBPA N1N2N3 by action of vWbp-activated FXIII. Mixtures incubated with dansyl cadaverine (lane1) or dansyl-ε-aminocaproyl-QQIV peptide (lane 2) were separated by SDS-PAGE and the gel was examined under ultraviolet light (**A**) and then stained with Bio-Safe Coomassie staining (**B**). The figures are representative of three independent experiments. Molecular masses of standard proteins are indicated on the left side of the panel. Arrows show the positions of FnBPA N1N2N3 domain.

Severina et al*.* demonstrated that thrombin-activated FXIII catalyses covalent cross-linking between full-length FnBPA and Fbg^43^. Along this line, we firstly examined the presence of reactive glutamine or lysine residues in FnBPA N1N2N3 (A domain) assessing the ability of vWbp-activated FXIII to incorporate specific amine-donor (dansylcadaverine) or acceptor (dansyl-ε-aminocaproyl-QQIV peptide) synthetic probe into the recombinant bacterial protein. The reaction products were analyzed by SDS-PAGE and revealed under ultraviolet light (Fig. 3A) prior to staining the gel with Coomassie Blue (Fig. 3B). By the image obtained under ultraviolet illumination it appeared that vWbp-activated FXIII catalysed the incorporation of dansylcadaverine (lane 1), but not of dansyl-ε-aminocaproyl-QQIV probe (lane 2) (Fig. 3A), suggesting the presence in the A domain of reactive glutamine but not lysine residues.

**Figure 4:**
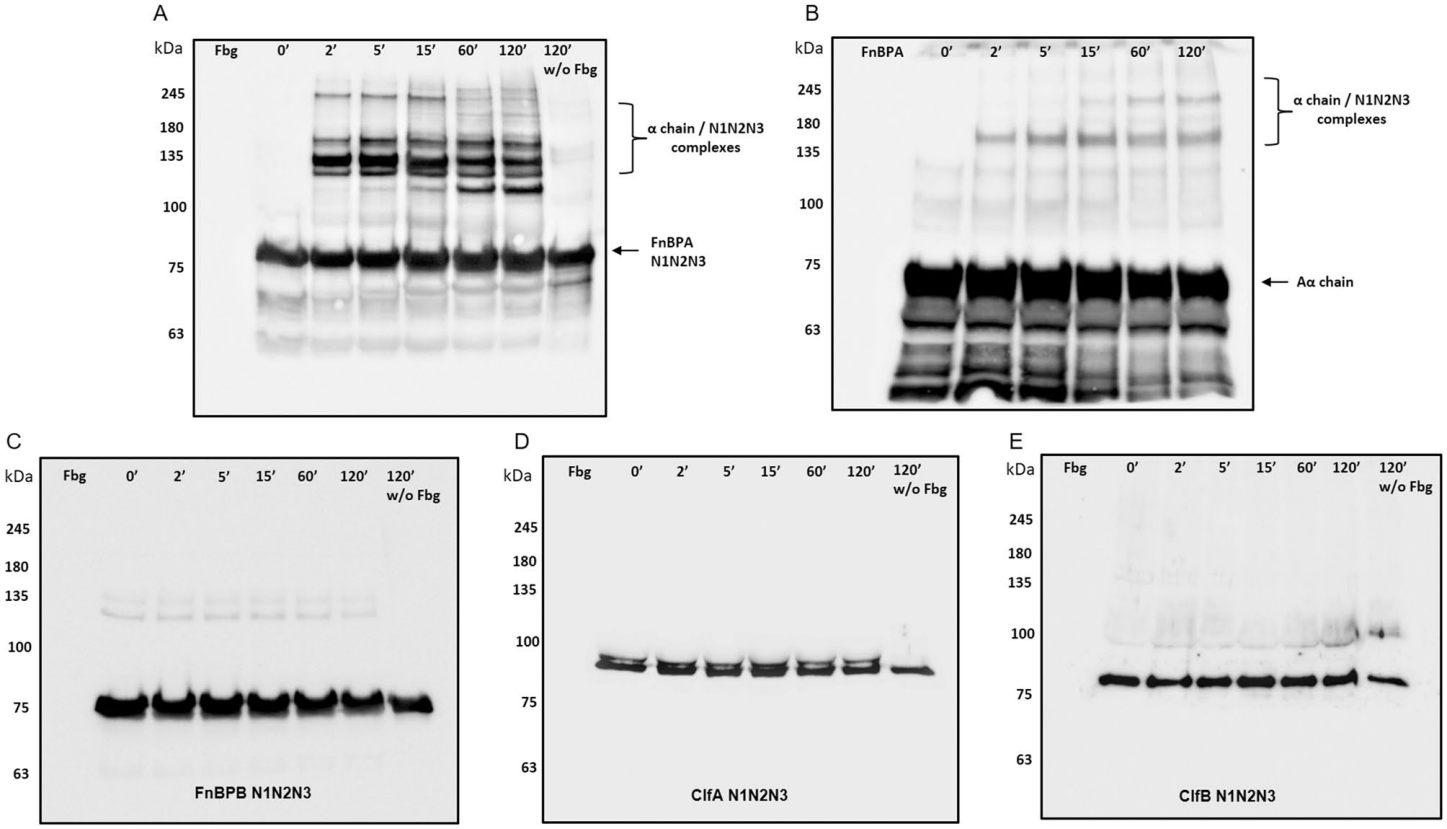
vWbp-activated FXIII elicits cross-links formation between region A of FnBPA and α-chain of Fbg. Panel (**A**) shows cross-linking of recombinant FnBPA N1N2N3 domain to Fbg by action of vWbp-activated FXIII. After indicated time intervals mixtures were separated by SDS-PAGE under reducing conditions and transferred to a PVDF membrane. The membrane was probed with anti-FnBPA IgG followed by secondary HRP-conjugated IgG. (**B**) Cross-links formation between FnBPA N1N2N3 domain and Fbg by vWbp-activated FXIII was analyzed by incubating the membrane with anti-α chain Fbg IgG followed by secondary HRP-conjugated IgG. Arrows show the positions of the α chain or α chain/FnBPA N1N2N3 complexes. (**C**-**E**) Cross-linking formation of the recombinant A domain of FnBPB (**C**), ClfA (**D**) or ClfB (**E**) with Fbg by vWbp-activated FXIII were analyzed incubating the PVD membranes with mouse FnBPB, ClfA or ClfB IgG, respectively, followed by secondary HRP-conjugated IgG. The figures are representative of three independent experiments. Molecular masses of standard proteins are indicated on the left side of each panel. Incubation times (0–120 min) on the top of each panel are also specified. Original blots are presented in Supplementary Figure 3.

To further investigate the formation of cross-links between FnBPA and Fbg the substrates were incubated with vWbp-activated FXIII for increased periods of time and the mixtures subjected to SDS-PAGE under reduced conditions and Western immunoblotting using a FnBPA antibody. In these conditions, the formation of high molecular complexes including FnBPA were observed (Fig. 4A). In those mixtures from which Fbg was omitted no high molecular mass complexes were detected. The specificity of the antibody as probe was further assessed by the absence of its reactivity with mixtures containing Fbg only. SDS-PAGE and immunoblotting of mixtures containing FnBPA/Fbg and activated FXIII with a Fbg α chain antibody revealed the production of high molecular weight heteropolymers made of FnBPA and the α-chain (Fig. 4B). No covalent incorporation of the γ chain in the complexes was observed when the mixtures were subjected to SDS-PAGE and nitrocellulose membrane was probed with a Fbg γ chain monoclonal antibody (data not shown).

The specific formation of the FnBPA/Fbg covalent complexes was indicated by the absence of high molecular weight products when A region of FnBPB, ClfA or ClfB proteins were individually incubated for increasing times with Fbg in the presence of vWbp-activated FXIII and then tested by SDS-PAGE and immunoblotting with appropriate antibody (Fig. 4C,D and E).

### N1 subdomain of region A of FnBPA is involved in cross-link formation with fibrin(ogen)

**Figure 5:**
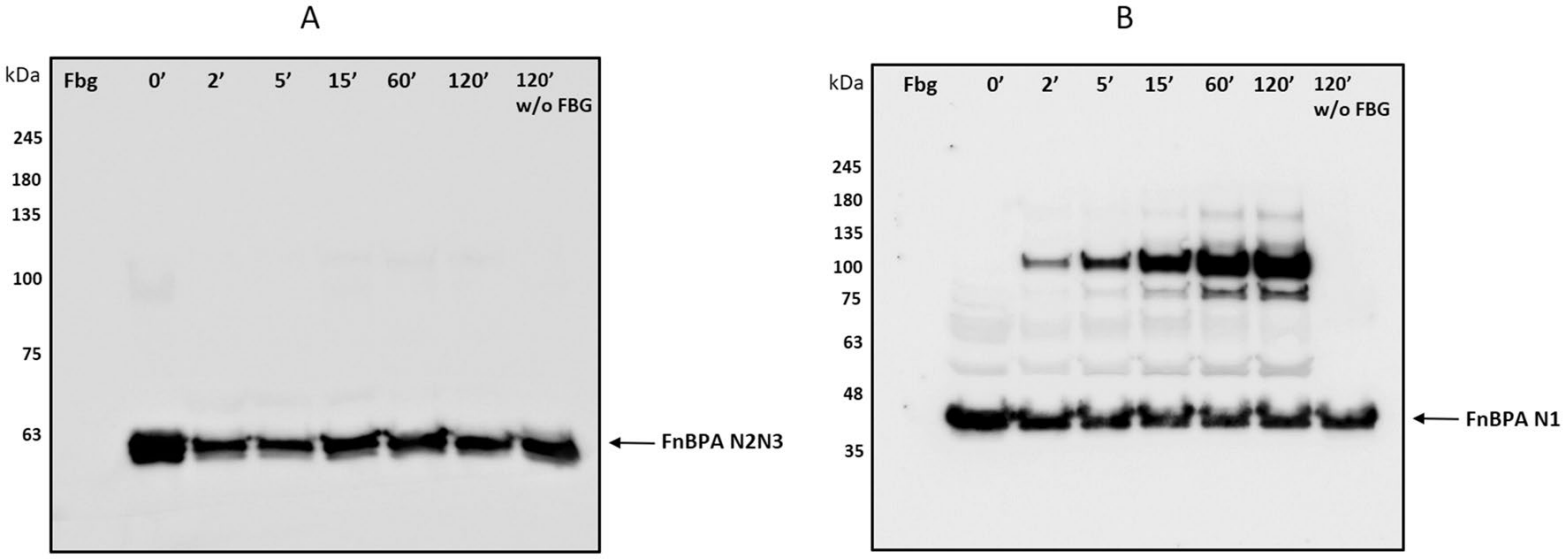
N1 subdomain of region A of FnBPA is involved in cross-link formation with Fbg. Mixtures containing FnBPA N2N3 (**A**) or FnBPA N1 (**B**) proteins and Fbg were incubated in the presence of vWbp-activated FXIII for the indicated times (top of each panel) and then separated by SDS-PAGE under reducing conditions and transferred to a PVDF membrane. Membranes were probed with FnBPA IgG followed by secondary HRP-conjugated IgG. Arrows show the positions of FnBPA N2N3 domain (**A**) or FnBPA N1 subdomain (**B**). The figures are representative of three independent experiments. Molecular masses of standard proteins are indicated on the left side of each panel. Original blots are presented in Supplementary Figure 4.

To localize the site in FnBPA N1N2N3 involved in cross-link formation, recombinant FnBPA N1 subdomain or FnBPA N2N3 protein were incubated for increasing times with Fbg in the presence of vWbp-activated XIII. Formation of covalent products was assessed subjecting the mixtures to SDS-PAGE and Western immunoblotting using a FnBPA antibody. No covalent complexes were detected incubating N2N3 protein with Fbg (Fig. 5A), while incubation of N1 subdomain with Fbg resulted in the formation of high mol weight complexes (Fig. 5B). Plausibly, the major heterocomplex (110 kDa) that accumulates over time is the result of cross-linking between the N1 subdomain (40 kDa) and the Fbg α chain (70 kDa).

### TG2 catalyses the formation of cross-links between FnBPA and Fbg

**Figure 6:**
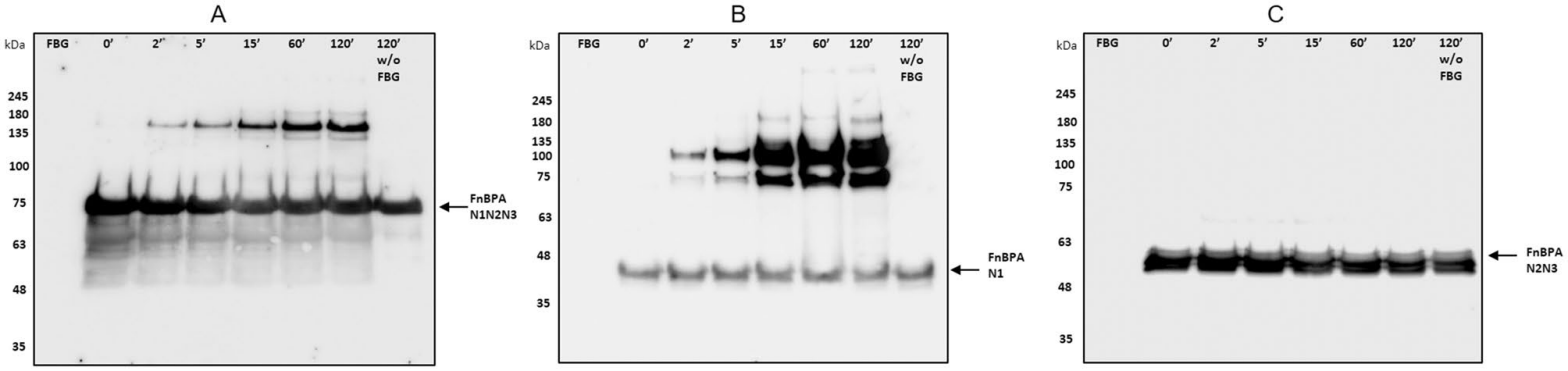
TG2 promotes formation of cross-links between N1 domain of FnBPA and Fbg. Products of cross-linking reactions between FnBPA N1N2N3 (**A**), N1 (**B**) and N2N3 (**C**) proteins and Fbg generated by action of TG2 were separated by SDS-PAGE under reducing conditions and transferred to a PVDF membrane. Membranes were probed with anti-FnBPA IgG followed by secondary HRP-conjugated IgG. Arrows show the positions of unreactive FnBPA proteins. The figures are representative of three independent experiments. Molecular masses of standard proteins are indicated on the left side of each panel. Incubation times (0–120) of the mixtures are indicated on the top of each panel. Original blots are presented in Supplementary Figure 5.

Transglutaminase 2 (TG2) is the most widely distributed and most abundantly expressed member of the transglutaminase family of enzymes^35^. In view of the fact that TG2 is also highly expressed in several tissues, we decided to investigate the catalytic role of the enzyme in the formation of covalent polymers between FnBPA and Fbg^35^. To this end, FnBPA N1N2N3, N1 or N2N3 proteins were incubated for increasing time intervals with Fbg in the presence of TG2. The incubation mixtures were then analysed by SDS-PAGE and Western immunoblotting using a FnBPA antibody. As reported in Fig. 6, covalent complexes between N1N2N3 and Fbg with size comparable to those obtained with activated FXIII were observed. Moreover, TG2 induced heteropolymers formation between N1 and Fbg, whereas no cross links were formed when N2N3 was incubated with Fbg.

### Interaction of *S. aureus* expressing FnBPA with Fbg is reinforced by the action of vWbp-activated FXIII

**Figure 7:**
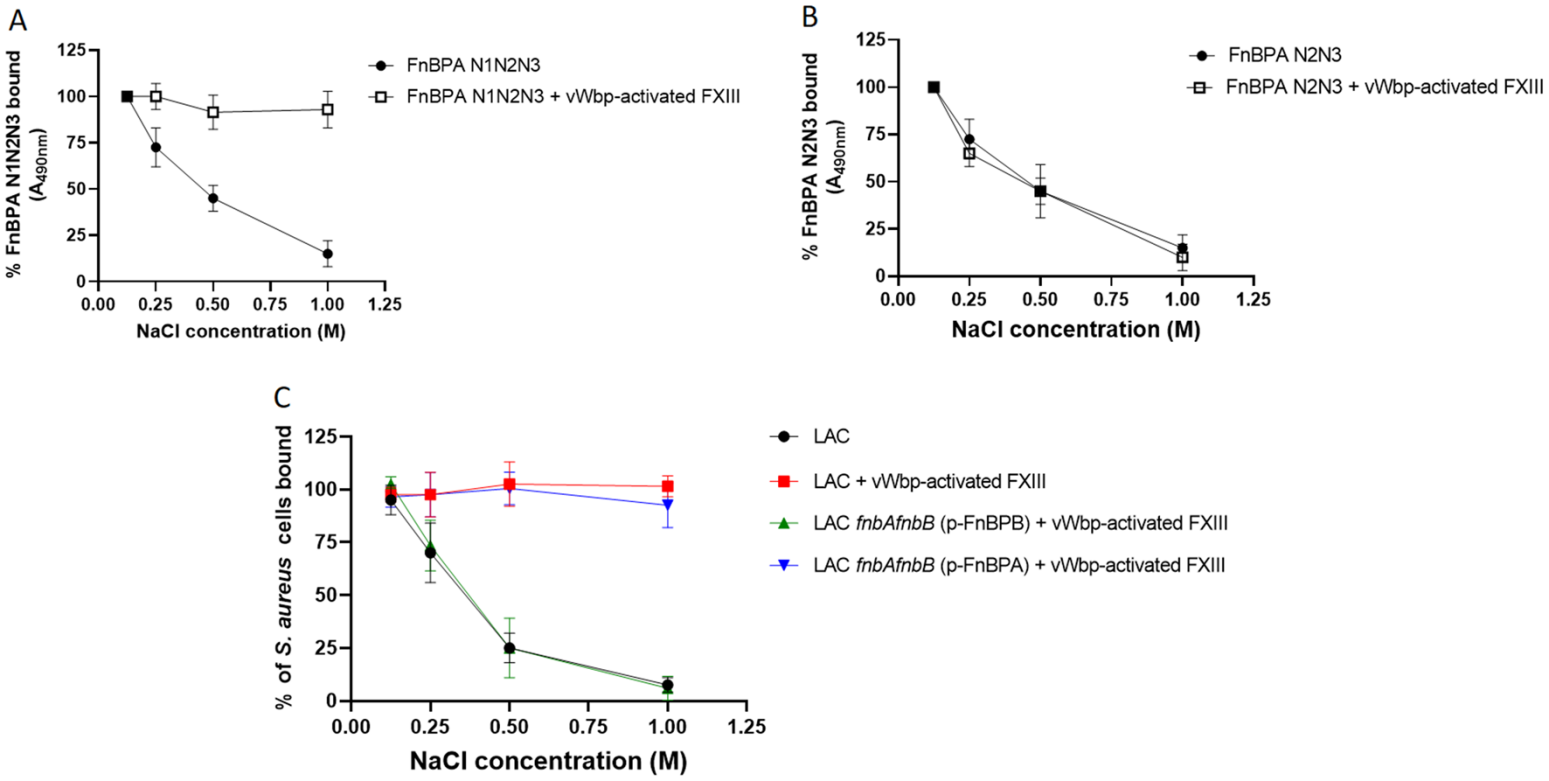
Cross-link formation induced by vWbp-activated FXIII reinforces binding between FnBPA and Fbg-coated surface. Mixtures of vWbp, ProT and FnBPA N1N2N3 (**A**) or FnBPA N2N3 (**B**) were incubated with immobilized Fbg in the presence/absence of FXIII. Wells were extensively washed with increasing concentrations of NaCl. Binding of FnBPA proteins to Fbg was detected by addition of a mouse FnBPA antibody followed by secondary HRP-conjugated IgG to the wells. (**C**) The effect of increasing concentrations of NaCl on the adhesion of *S. aureus* LAC wt cells (red) or fnbAfnbB double mutant cells overexpressing FnBPB (green) or FnBPA (blue) to Fbg was evaluated by incubating Fbg-coated microtiter wells with a mixture of vWbp, ProT and bacterial cells in the presence of FXIII. Wells were then washed with the indicated concentrations of NaCl and the attached bacterial cells detected with crystal-violet. The effect of salt concentrations on *S. aureus* LAC adhesion in absence of FXIII is also shown (black). Adhesion levels are expressed as a percentage of controls, where washings of the wells were performed with 150 mM NaCl. Data are expressed as means ± S.D. of triplicate tests.

Next, we evaluated the potential role of vWb-activated FXIII on the stabilization of FnBPA-mediated adhesion of *S. aureus* LAC cells to Fbg. Preliminarily, the N1N2N3 and the N2N3 recombinant proteins were allowed to interact with surface-coated Fbg in the presence of vWbp-activated FXIII in an ELISA assay. Then, the wells were washed with increasing concentrations of NaCl (0.15–1.0 M) and then tested for ligand binding to the immobilized substrate. Assessment of Interaction of N1N2N3 protein with Fbg the presence of activated FXIII showed a complete resilience to ionic strength. Conversely, due to its inability to form covalent bonds with Fbg, N2N3 protein dissociates from Fbg as the ionic strength increases (Fig. 7A and B). For incubations where activated FXIII was omitted, a progressive, substantial reduction of ligand binding to Fbg was observed.

To investigate the stabilizing effect of covalent cross links on the attachment of *S. aureus* LAC to Fbg, microtiter wells coated with Fbg were incubated with bacterial cells in the presence /absence of vWbp-activated FXIII. Wells were then treated with increasing concentrations of NaCl and tested for bacterial adhesion. As expected, only bacteria incubated with activated FXIII showed resilience to the ionic strength. In confirmation of this, cells of the double mutant *S. aureus* LAC *fnbAfnbB* overexpressing FnBPA incubated with vWbp-activated FXIII showed complete resistance to the ionic strength, while cells of the double mutant *fnbAfnbB* expressing high levels of FnBPB demonstrated susceptibility to the ionic strength and inability to remain attached to Fbg. (Fig. 7C).

## Discussion

Detailed studies on the cross-linking between FnBPA and Fbg and Fn promoted by thrombin-activated FXIII has been performed by several authors^43–45^. It has proposed that transglutaminase activity of activated FXIII is likely to serve as a mechanism to incorporate *S. aureus* cells at site of vascular injury and to establish infection^46,47^. Thrombin-activation of FXIII elicits cross-linking in other bacterial species as well. For example, *Streptococcus pyogenes* cells can be covalently incorporated inside the clot via cross-linking of bacteria to fibrin fibers^48^. In Group B *Streptococcus* (GBS), thrombin-activated FXIII promotes bacterial entrapment within fibrin clots by cross-linking fibronectin to ScpB, a GBS Fn-binding surface protein^49^. Although thrombin-activated FXIII has a primary role in the covalent cross-linking of bacteria to Fbg and fibronectin, incorporation of FnBPA-mediated staphylococcal cells into the clots may also depend on the FXIII activation by secreted staphylococcal coagulases Coa and vWbp. In fact, Coa and vWbp act as nonproteolytic activators of ProT that in turn converts Fbg to fibrin and FXIII precursor into activated FXIII^27^. Thus, the production of coagulases such as vWbp may implement the pathogenetic potential of this bacterium. In view of these considerations, we investigated the role of vWbp-activated FXIII in the formation of cross-links between Fbg and FnBPA.

Under in vitro conditions we showed that the vWbp is mostly produced in the exponential phase of bacterial growth and that the protein is expressed in vivo and is immunogenic. We also found that secreted vWbp can rebind to the bacterial surface and found that this anchoring is mediated by PPG and LTA. Due to the different structure of PPG and LTA, it is plausible that two distinct binding sites exist on vWbp molecule. Our results also indicate that, when bound to the cell surface, vWbp recruits and activates ProT on *S. aureus* surface, possibly generating active FXIII and cross-linking of Fbg to surface-associated FnBPA.

A study performed incubating vWbp-activated FXIII with FnBPA N1N2N3 region in the presence of fluorescent probes dansyl-cadaverine or glutamine-containing peptide revealed the presence of reactive Gln residues in the targeted substrate. Differently than previously reported, no Lys residues potentially involved in cross-link formation were observed in this domain^43^.vWbp-activated FXIII catalyses the formation of intermolecular ε(γ-glutamyl) lysine isopeptide bonds between Fbg and the A region (N1N2N3 domains) of FnBPA. Moreover, the cross-linking site for Fbg in FnBPA was localized in N1 subdomain, which contains the functional reactive Gln103 (Q) residue, previously identified as the main lysine acceptor residue within Fbg^43^. On the other hand, no cross-linking was observed when incubating the recombinant FnBPA N2N3 domains with Fbg. In line with the work performed by Severina et al*.*^43^*,* we also found that the acceptor glutamine site involved in cross-linking reaction was localized in the α chain of Fbg.

Thus, interaction between Fbg and full length N1N2N3 occurs a) through the non-covalent binding of the Fbg γ chain to the FnBPA N2N3 region via the dock, lock and latch (DLL) mechanism^19,20^ and b) via the covalent interaction of the Fbg α chain with the N1 subdomain.

No isopeptide bond was observed when region A of other Fbg-binding proteins such as ClfA, ClfB or FnBPB was tested for cross-linking reactions in the presence of vWbp-activated FXIII. In accordance with this, multiple alignments of the N-terminal sequence of ClfA, ClfB, FnBPB and FnBPA showed that none of the homologous sequences of ClfA, ClfB and FnBPB adhesins centered on the Gln103 of N1 subdomain of FnBPA possesses a reactive Gln103 residue (Fig. S2), Hence, Gln103 is essential to establish a covalent cross-link with Fbg.

After that, we wondered whether other tranglutaminase family members, such as the most ubiquitously expressed tissue TG2^35^, could have a role in promoting cross-linking of Fbg to FnBPA.

Incubation of N1N2N3 region with Fbg in the presence of TG2 resulted in a rapid and efficient formation of high molecular covalent complexes, with a kinetics similar to that reported when the cross-linking of N1N2N3 to Fbg was examined in the presence of activated FXIII. Of note, as reported for the reaction catalysed by activated FXIII, the complex formation promoted by TG2 involved the N1 subdomain. Thus, although operational in different contexts and exhibiting different biochemical and regulatory properties, the transglutaminase activities of FXIII and TG2 generate almost identical bioproducts.

We also tested whether vWbp-activated FXIII can catalyse covalent attachment and incorporation of *S. aureus* to Fbg. To this end, we primarily demonstrated that the covalent FnBPA/Fbg complex can be formed in the presence of activated FXIII and does not dissociate even in the presence of high levels of ionic strength.

Differently from a mutant strain of *S. aureus* LAC overexpressing FnBPB, the *S. aureus* LAC wt and its mutant overexpressing FnBPA, when incubated with Fbg in the presence of activated FXIII and then treated with increasing amounts of NaCl, showed resilience to ionic strength. Overall, these results indicate that activated FXIII not only elicits a covalent complex formation between recombinant FnBPA and Fbg, but also promotes FnBPA-mediated cross-linking of *S. aureus* cells to immobilized Fbg. Thus, the covalent attachment of bacteria to Fbg induced by activated FXIII may represent a host innate defence mechanism to diminish their dissemination and invasion^48^. Alternatively, covalent entrapment of *S. aureus* cells expressing FnBPA within fibrin fibers may be a prerequisite for the persistence of staphylococci in clots and a potential mechanism to escape the immune defence system^26^.

The methodological approach described above may be useful to extend the value of covalent cross-links formation between additional bacterial and host components to stabilize tissue colonization and infection. The wealth of *S. aureus* surface proteins and their host ligands could be the natural candidates of future studies.

This study reconfirms the central role of FnBPA at the interface between *S. aureus* and the colonized host. In consideration of previous work demonstrating the formation of covalent bonds between the repetitive region of FnBPA and fibronectin by thrombin-activated FXIIII^44^, a further analysis of the molecular aspects of cross-linking of FnBPA to fibronectin may be worth exploring.

The biological significance and the importance in pathogenesis of Fbg cross-linking to FnBPA are still the subject of speculation. We can envisage at least two possible scenarios : i) cross linking reactions could have a role in limiting dissemination of *S. aureus* from the site of infection, such as fibrin clots, to the whole organism minimizing the events that progress to sepsis. In support of this hypothesis, it has been demonstrated that mice infected with GBS exhibit a containment of the bacterial infection, whereas mice deficient in FXIII show a greater susceptibility to systemic infection. Consistent with this observation, administration of exogenous FXIII to mutated mice reduced bacterial spreading and dissemination^49^. Therefore, the catalytic action of transglutaminases could be considered as part of innate defence mechanisms of the host. ii) covalent heteropolymers formed by Fbg and FnBPA through the enzymatic action of vWbp-activated FXIII or TG-2 could contribute to the stabilization of *S. aureus* adherence to the extracellular matrix of tissues constitutively expressing Fbg^50–52^ and to Fbg-rich sites such as vegetations in a host damaged cardiac valve or fibrin(ogen)-coated biomaterials. Thus, through the action of transglutaminases, the bacterium could opportunistically manipulate the host defence mechanisms to its own benefit.

Studies are in progress in our laboratory to determine the value of such important issues.

## Methods

### Bacterial strains and culture conditions

All strains used in this study are listed in Table 1. *S. aureus* cells were grown overnight in brain heart infusion (BHI) (VWR International Srl, Milan, Italy) at 37 °C with shaking. *Escherichia coli* BL21 (DE3) (Invitrogen, Carlsbad, CA, USA) transformed with vector pQE30 or pET22b (Integrated DNA Technologies, Leuven, Belgium) was grown in Luria agar and Luria broth (VWR International Srl) containing 100 μg/ml ampicillin (Sigma Aldrich) at 37 °C. In those experiments, where a defined number of cells were used, bacteria were harvested from the cultures by centrifugation, washed, suspended in phosphate-buffered saline (PBS), and counted in a Petroff-Hausser chamber.

**Table 1:** List of bacterial strains used in this study.

Bacterial strain	Relevant properties	Reference
*S. aureus*		
LAC wt	Community-associated MRSA of USA300 lineage	^53^
LAC *spa*	Strain derivative of LAC* deficient in protein A; constructed by transduction of *spa*::Kan^r^ by phage 85 into strain LAC*	^54^
LAC *srtA*	Constructed by the transduction of *srtA*::Erm^r^ from Newman *srtA* using bacteriophage 85	^11^
LAC *fnbAfnbB* (pFnBA)	*fnbA fnbB* double mutant transformed with plasmid-expressing FnBPA	^55^
LAC *fnbAfnbB* (pFnBB)	*fnbA fnbB* double mutant transformed with plasmid-expressing FnBPB	^55^
*E. coli*		
BL21 (DE3)	*E. coli* cloning host	Invitrogen

Kan^r^, kanamycin resistance; Erm^r^, erythromycin resistance.

### Plasmid and DNA manipulation

The DNA fragments encoding the N1 (residues 37–193) and N1-3 (residues 37–511) regions of FnBPA were PCR amplified from the genome of *S. aureus* strain 8325–4 using the forward primer 37-193For (5’-GCATCACCATCACCATCACGGATCCGCATCAGAACAAAAGACAAC) for both constructs and the reverse oligonucleotides 37–193 (5’-TAATTAAGCTTGGCTGCAGGTCGACCTACGTTTCCACTTTCGCGTTAC) for N1 region or 37–511 (5’-TAATTAAGCTTGGCTGCAGGTCGACCTAATTTTTCTCATTTCCGTTC) for N1-3 region. Amplified fragments were cloned by Gibson assembly (NEBuilder HiFi DNA Assembly Cloning Kit, New England Biolabs) into the expression plasmid pQE30 (Qiagen), between the BamHI and SalI restriction sites, thus generating constructs pFnBPAN1 and pFnBPAN1-3. All constructs were verified by sequencing using primers pQEfor (5’-GTATCACGAGGCCCTTTCGTCT) and pQErev (5’-CATTACTGGATCTATCAACAGGAG). Cloning of N2N3 was performed as previously reported^9^.

### Expression and purification of recombinant proteins

Recombinant FnBPA N1N2N3, and N1 regions were expressed from pQE30 (Millipore-Sigma, MA, USA) in *E. coli* BL21 (DE3)). Overnight starter culture was diluted at 1:40 in Luria broth containing ampicillin and incubated with shaking until the culture reached the exponential phase (OD_600nm_ = 0.4–0.6). Recombinant protein expression was induced by the addition of 1 mM (final concentration) isopropyl 1-thio-β-D-galactopyranoside (IPTG) (Sigma-Aldrich) to the culture and incubated over-night at 28 °C. Bacterial cells were harvested by centrifugation and frozen at − 80 °C. Cells were re-suspended in lysis buffer (50 mM NaH_2_PO_4_, 300 mM NaCl, pH 8.0 supplemented with 1 mM MgCl_2_) containing 1 mM phenyl-methanesulfonyl-fluoride (PMSF) (Sigma-Aldrich) and 20 μg/mL of protease-free DNase I (Sigma-Aldrich) and lysed by sonication (70% amplitude, 12 × 30″ on/off, 1′30″ interval between sonication steps). The cell debris was removed by centrifugation and proteins purified from the supernatants by Ni^+2^-affinity chromatography on a HiTrap chelating column (GE Healthcare, Buckinghamshire, UK). Protein purity was assessed by SDS-PAGE and Bio-Safe Coomassie staining (BioRad, Hercules, CA, USA). A bicinchoninic acid protein assay (Pierce, Rockford, IL, USA) was used to measure the concentration of purified proteins.

Recombinant proteins FnBPA N2N3^9^, FnBPB N1N2N3^8^, ClfA N1N2N3^56^, ClfB N1N2N3^57^ and vWbp^27^ were expressed with His_6_ N-terminal affinity tags and purified as reported above.

### Reagents, proteins and antibodies

BSA (bovine serum albumin), skim milk and dansylcadaverine were purchased from Sigma-Aldrich. Dansyl-ε-aminocaproyl-QQIV was synthesized by GenScript (Rijswijk, Netherlands). Human prothrombin, human factor XIII (FXIII) and Fbg were purchased by Prolytix (VT USA). Ultrapure peptidoglycan (PPG) and lipoteichoic acid (LTA) from *S. aureus* were purchased by InvivoGen (CA, USA). The chromogenic substrate S2238 (D-Phe-Pip-Arg-pNA) was obtained from Chromogenix (Milan, Italy). Translutaminase-2 (TG2) was obtained from Zedira GmbH (Darmstadt, Germany). IgG from patients with infective endocarditis were isolated as previously reported^40^. IgG collection was carried out in accordance with relevant guidelines and regulations; all experimental protocols were approved by the ethical board of the University of Pavia and informed consent was obtained from all human participants. vWbp, FnPBA, FnBPB, ClfA and ClfB polyclonal antibodies were raised in mice by routine immunization procedure using each purified bacterial protein as antigen. The study is reported in accordance with ARRIVE guidelines (https://arriveguidelines.org). The polyclonal antibodies production method was carried out in accordance with relevant guidelines and regulations; all experimental protocols were approved by the ethical board of the University of Pavia. Monoclonal rabbit anti-α chain and anti-γ chain antibodies were purchased from Bioss Antibodies (MT, USA). Rabbit anti-mouse, rabbit anti-human, goat anti-rabbit horseradish peroxidase (HRP)-conjugated secondary antibodies were purchased from Dako Cytomation (Glostrup, Denmark). Avidin-peroxidase was purchased from Sigma Aldrich. OPD tablets (*o*-phenylenediamine dihydrochloride) were purchased by ThermoScientific (Rockford, IL, USA).

### vWbp expression during bacterial growth phases

The expression of vWbp during different bacterial growth phases was assessed through an ELISA assay. For this purpose, cells of a mutated strain deleted of protein A (*S. aureus* LAC *spa*) were grown for the indicated times. Bacteria were harvested by centrifugation and culture supernatants filtered (pore size 0,22 μm). 100 μl of individual supernatants were immobilized onto microtiter wells overnight at 4 °C. The wells were washed three times with 0.5% (v/v) Tween 20 in PBS (PBST) and treated for 1 h at 22 °C with 2% (v/v) BSA in PBS. The binding of vWbp to the surface-coated material was detected incubating the wells for 1 h with a mouse polyclonal anti-vWbp IgG (1:2000) in 1% (v/v) BSA followed by an HRP-conjugated anti-mouse IgG (1:1000) in 1% (v/v) BSA for 45 min. After washing, OPD was added to the wells and the absorbance at 490 nm measured in an ELISA plate reader (Bio-Rad) The bacteria growth curve was obtained evaluating the bacterial density at 600 nm.

### Reactivity with vWbp of IgG from patients with infective endocarditis

To test the reactivity of IgGs obtained from sera of patients with endocarditis, recombinant vWbp was immobilized onto microtiter wells (1 µg/well). After blocking with BSA, the wells were incubated with IgG (1 μg/well) from patients and healthy donors. The binding of IgG was revealed by the addition to the wells of an HRP-conjugated polyclonal rabbit anti‐human IgG (1:1000) and the absorbance was determined as reported above.

### Biotin labelling of vWbp

Biotin labelling of vWbp was carried out using a Biotin Conjugation Kit (Fast, Type A) (Abcam, Cambridge, MA, USA). Labelled sample was dialysed and the protein concentration measured using a bicinchoninic acid protein assay (Pierce).

### Rebinding of vWbp to the bacterial surface

To measure rebinding of vWbp to the bacterial surface, microtiter wells were coated overnight at 37 °C with 100 μl of *S. aureus* LAC wt suspensions (OD_600nm_ = 1.0) in PBS from both stationary and exponential phases. After blocking the wells with BSA, the plates were incubated for 1 h with increasing concentrations of biotinylated vWbp (from 0 to 10 μg/well). After several washings, the wells were treated with avidin-peroxidase (1:1000) and the absorbance determined as reported above. Cells of *S. aureus* LAC *srtA* from the stationary phase of growth were tested for vWbp rebinding in the conditions reported above.

### Effect of increasing concentration of NaCl on the interaction of recombinant FnBPA or of *S. aureus* cells with Fbg

To assess the effect of ionic strength on the binding of recombinant FnBPA N1N2N3 or N2N3 proteins to immobilized Fbg, microtiter wells were coated overnight with 5 μg/well of Fbg. After blocking with BSA, the wells were added with a mixture of vWbp (200 nM), ProT (200 nM), FXIII (20 nM) and FnBPA N1N2N3 or N2N3 (1.5 μM) in presence of 5 mM CaCl_2_ and incubated for 2 h at 37 °C. After washing with PBST, the wells were extensively washed with increasing concentration of NaCl (from 0,15 to 1 M) and bound FnBPA detected with a mouse polyclonal anti-FnBPA IgG (1:1000) followed by HRP-conjugated rabbit anti-mouse IgG (1:1000). To measure attachment of *S. aureus* cells to Fbg and the effect of ionic strength on the interaction, Fbg-coated wells were incubated for 2 h at 37 °C with 1 × 10^8^ cells of *S. aureus* strain LAC wt or the double mutant *fnbAfnbB* transformed with plasmid overexpressing FnBPB or FnBPA in the presence of vWbp-activated FXIII. After treatment with increasing concentration of NaCl, bacteria attached to the wells were fixed with formaldehyde (25% v/v), stained with crystal violet (0.5% w/v) and the absorbance at 595 nm determined using an ELISA plate reader.

### Activation of ProT by soluble or bacteria-bound vWbp

Activation of human ProT by vWbp was performed incubating human ProT (0.1 mg/ml, 1.38 μM) with equimolar concentration of vWbp in presence of 20 μM S2238 (D-Phe-Pip-Arg-pNa) in TBS-CaCl_2_ (25 mM Tris–HCl pH 7.8, 0.15 M NaCl, 5 mM CaCl_2_) for 2 h at 37 °C. The absorbance of released pNa (p-Nitroaniline) was determined at 405 nm using an ELISA plate reader. Experiments performed with mixtures where ProT or vWbp was omitted were used as control.

The ability of bacteria-associated vWbp to activate ProT, was determined coating microtiter wells with 100 µl of *S. aureus* LAC suspensions (OD_600nm_ of 1.0) at 37 °C. The plates were incubated with 10 µg/well of vWbp for 1 h and then added with ProT (0.1 mg/ml, 1.38 μM) in presence of 20 μM S2238 in TBS-CaCl_2_ for 2 h at 37 °C. The absorbance of released pNA was determined as reported above. Wells coated with bacteria without vWbp and incubated with ProT were used as control. An additional control experiment was performed incubating immobilized bacteria/vWbp complexes with S2238 alone.

### Dot blot and western blot assays

#### Binding of vWbp to peptidoglycan and lipoteichoic acid

To evaluate the binding of vWbp to PPG and LTA, 5 μg of each compound was dotted onto a PVDF membrane (BioRad). After overnight incubation at 4 °C with 5% skim milk (w/v) in PBST, the membrane was treated for 1 h at 22 °C with 2 µg/mL of vWbp. The membrane was incubated with a mouse polyclonal vWbp antibody (1:5000) in 2% (w/v) skim milk for 1 h at 22 °C. Following several washings with PBST, the membrane was treated with 45 min at 22 °C with an HRP-conjugated rabbit anti-mouse IgG (1:1000) in 2% (w/v) skim milk and dot blots developed using the Westar Supernova detection kit (Cyanagen srl, Bologna, Italy). An ImageQuant™ LAS 4000 mini-biomolecular imager (GE Healthcare) was used to capture images of the spots. The signal intensities were quantified with ImageJ and plotted on GraphPad Prism.

#### Cross-linking of recombinant *S. aureus* proteins to Fbg promoted by vWbp-activated FXIII

To evaluate FnBPA, FnBPB, ClfA and ClfB cross-linking to fibrin(ogen) in the presence of vWbp-activated FXIII, Western immunoblotting assays were performed. Mixtures containing 200 nM ProT, 200 nM vWbp, 15 µg/ml FXIII, 5 µM Fbg and 2.5 µM of each bacterial protein were incubated at 37 °C in 25 mM Tris–HCl buffer, pH 7.5 containing 5 mM CaCl_2_ for the indicated times. Reactions were stopped by addition to the mixtures of a buffer containing 62.5 mM Tris–HCl, pH 6.8, 4 M urea, 2% SDS, 10% glycerol, 5% β-mercaptoethanol and 0.01% bromophenol blue. Samples were heated at 95 °C for 10 min, separated by 4.8–10% polyacrylamide gels SDS-PAGE and electroblotted onto a PVDF membrane. After overnight blocking with 5% (p/v) skim milk and washings, the membranes were immunostained with the appropriate mouse antibody against each the bacterial protein. The membranes were treated with an HRP-conjugated rabbit anti-mouse IgG (1:10,000), and the blots developed as reported above.

Detection of the presence of α or γ chain in heteropolymers was determined by using a monoclonal rabbit anti-α chain or anti- γ chain (1:5000) primary antibody and a goat anti-rabbit HRP-conjugated secondary antibody (1:10,000).

#### Cross-linking of FnBPA proteins to Fbg promoted by TG2

To evaluate isopeptide formation between FnBPA protein and Fbg promoted by the catalytic action of TG2, 2.5 µM of each recombinant FnBPA domain (N1N2N3, N2N3 or N1) was incubated with 5 µM Fbg in the presence of 50 nM TG2 in 25 mM Tris–HCl buffer, pH 7.5 containing 5 mM CaCl_2_ for the indicated times. Samples were heated at 95 °C for 10 min, separated by 4.8–10% polyacrylamide gels SDS-PAGE and electroblotted onto a PVDF membrane. After overnight blocking with 5% (p/v) skim milk, membranes were washed and immunostained with a FnBPA mouse polyclonal antibody diluted 1:5000. The bound antibody was detected by treatment of the membrane with an HRP-conjugated rabbit anti-mouse IgG (1:10,000).

### Incorporation of dansylcadaverine and dansyl-ε-aminocaproyl-QQIV into FnBPA by vWbp-activated FXIII

To investigate the covalent incorporation of synthetic probes dansylcadaverine and dansyl-ε-aminocaproyl-QQIV into FnBPA, mixtures containing 2 nM FnBPA, 200 nM ProT and 200 nM vWbp were incubated with 500 μg/mL FXIII in the presence of either 2 mM of dansylcadaverine or 2 mM of dansyl-PGGQQIV for 20 min at 37 °C in 20 mM TBS buffer, pH 7.4 containing 150 mM NaCl supplemented with 5 mM dithiothreitol and 5 mM CaCl_2_. Reactions were stopped by addition of 2% SDS and 10% β-mercaptoethanol. Controls were performed incubating the above mixtures in the presence of 2 mM EDTA. Samples were analyzed by SDS − PAGE and gels examined under ultraviolet light and then stained with Bio-Safe Coomassie staining.

### Statistical methods

Analyses were performed using Prism 4.0 (GraphPad). A minimum of three biological replicates (three independent experiments) were conducted for each experiment. Comparison of more than two groups were performed with the one-way ANOVA followed by Dunnett’s test. The two-tailed Student’s t-test was employed to compare two groups. *P* values < 0.05 were considered statistically significant, he following symbols are used, *,*P* < 0.05, **P < 0.01, ***P < 0.001.

## Supplementary Information

Supplementary Figure 1.

Supplementary Figure 2.

## Data Availability

The datasets generated or analyzed during the current study are available from the corresponding author on reasonable request.
