# Supplementary material for: von Willebrand factor-binding protein (vWbp)-activated factor XIII and transglutaminase 2 (TG2) promote cross-linking between FnBPA from Staphylococcus aureus and fibrinogen
Source: Sci Rep. 2023 Jul 19;13:11683. doi: 10.1038/s41598-023-38972-3 (PMC10356753; doi:10.1038/s41598-023-38972-3)
Supplement: Supplementary file 1 — Supplementary Figure 1. [file 41598_2023_38972_MOESM1_ESM.docx]

**Supplementary Fig. 1** Binding of increasing concentrations of biotinilated vWbp to *S. aureus* LAC wt or LAC derivative *srtA* deletion mutant cells immobilized onto microtiter wells was determined by addition of avidin-peroxidase. Data are expressed as means ± S.D. of triplicate tests

**Supplementary Fig. 2***.* ClfB, ClfA, FnBPA and FnBPB amino acid sequence alignments of part of the N1 subdomain surrounding Gln103 residue (Q highlighted in yellow) of FnBPA.
