# Supplementary material for: von Willebrand factor-binding protein (vWbp)-activated factor XIII and transglutaminase 2 (TG2) promote cross-linking between FnBPA from Staphylococcus aureus and fibrinogen
Source: Sci Rep. 2023 Jul 19;13:11683. doi: 10.1038/s41598-023-38972-3 (PMC10356753; doi:10.1038/s41598-023-38972-3)
Supplement: Supplementary file 2 — Supplementary Figure 2. [file 41598_2023_38972_MOESM2_ESM.pdf]

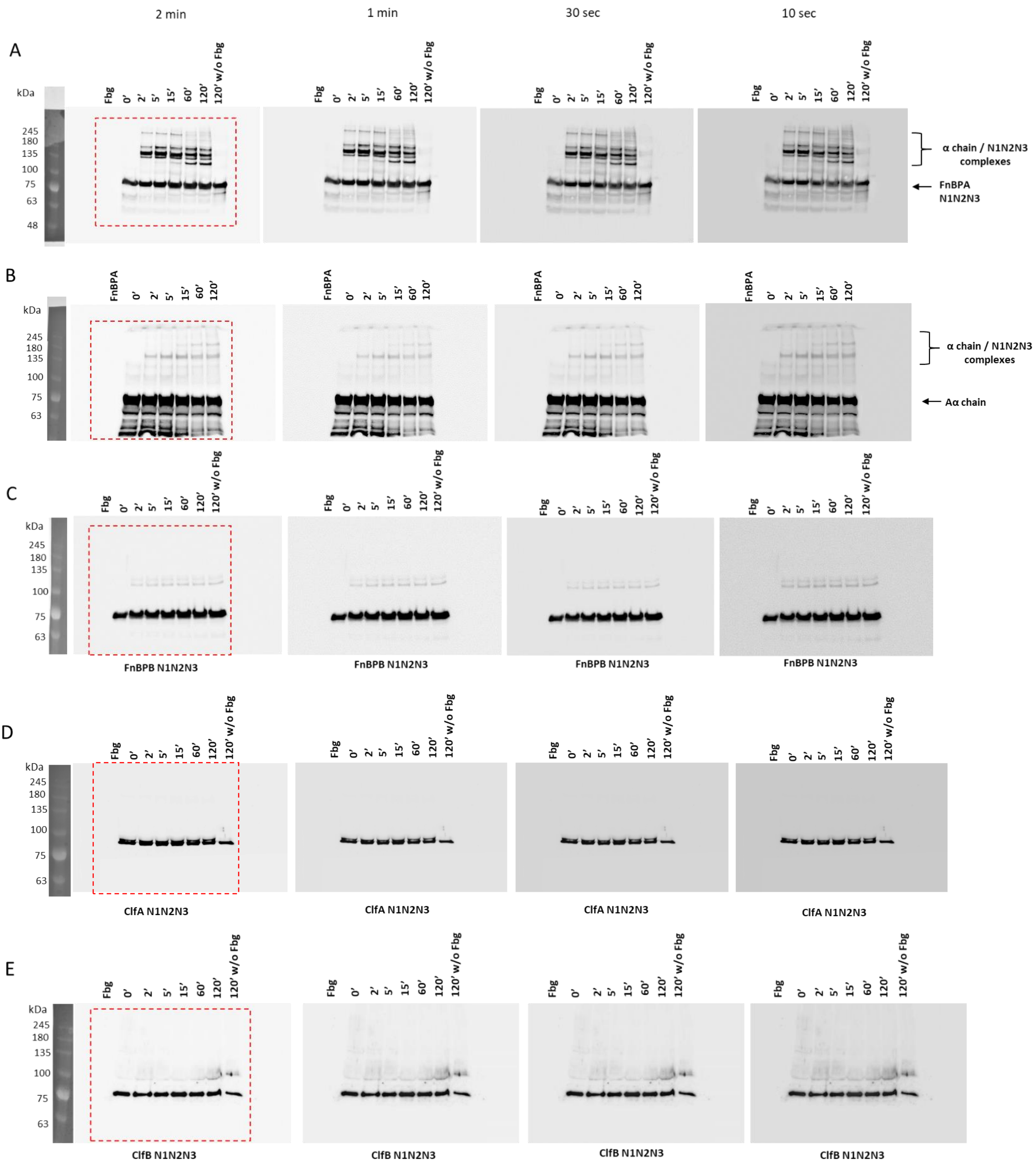

Supplementary Fig. 3. Full-length original Western blots shown in Fig. 4 of the main text are reported. The membranes were slightly cropped on the edges to make the results shown in Fig. 4 easier to understand. The area of each cropped membrane is indicated by a red dashed line. Multiple exposure times are reported on the top of the figure. The blots were not cut prior to hybridisation with antibodies. The standard protein size markers with the expected molecular weight are reported on the left. Incubation times on the top of each panel are also specified. Arrows show the positions of the α chain or α chain/FnBPA N1N2N3 complexes (A-B). Recombinant domains analysed are reported at the bottom of each panel (C, D and E).

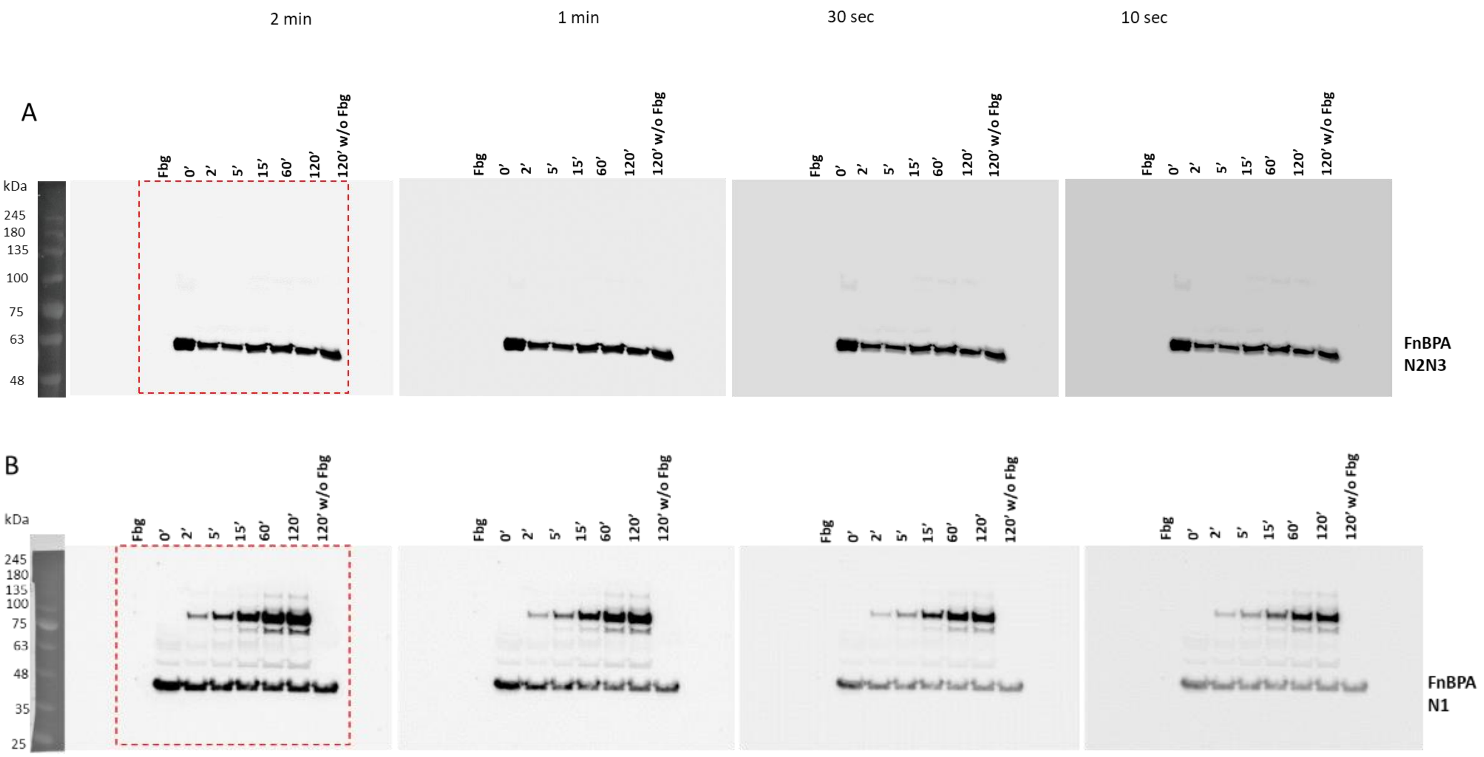

Supplementary Fig. 4. Full-length original Western blots shown in Fig. 5 of the main text are reported. The membranes were slightly cropped on the edges to make the results shown in Fig. 5 easier to understand. The area of each cropped membrane is indicated by a red dashed line. Multiple exposure times are reported on the top of the figure. The blots were not cut prior to hybridisation with antibodies. The standard protein size markers with the expected molecular weight are reported on the left. Incubation times on the top of each panel are also specified. Recombinant domains analysed are reported on the right of the panel (A and B).

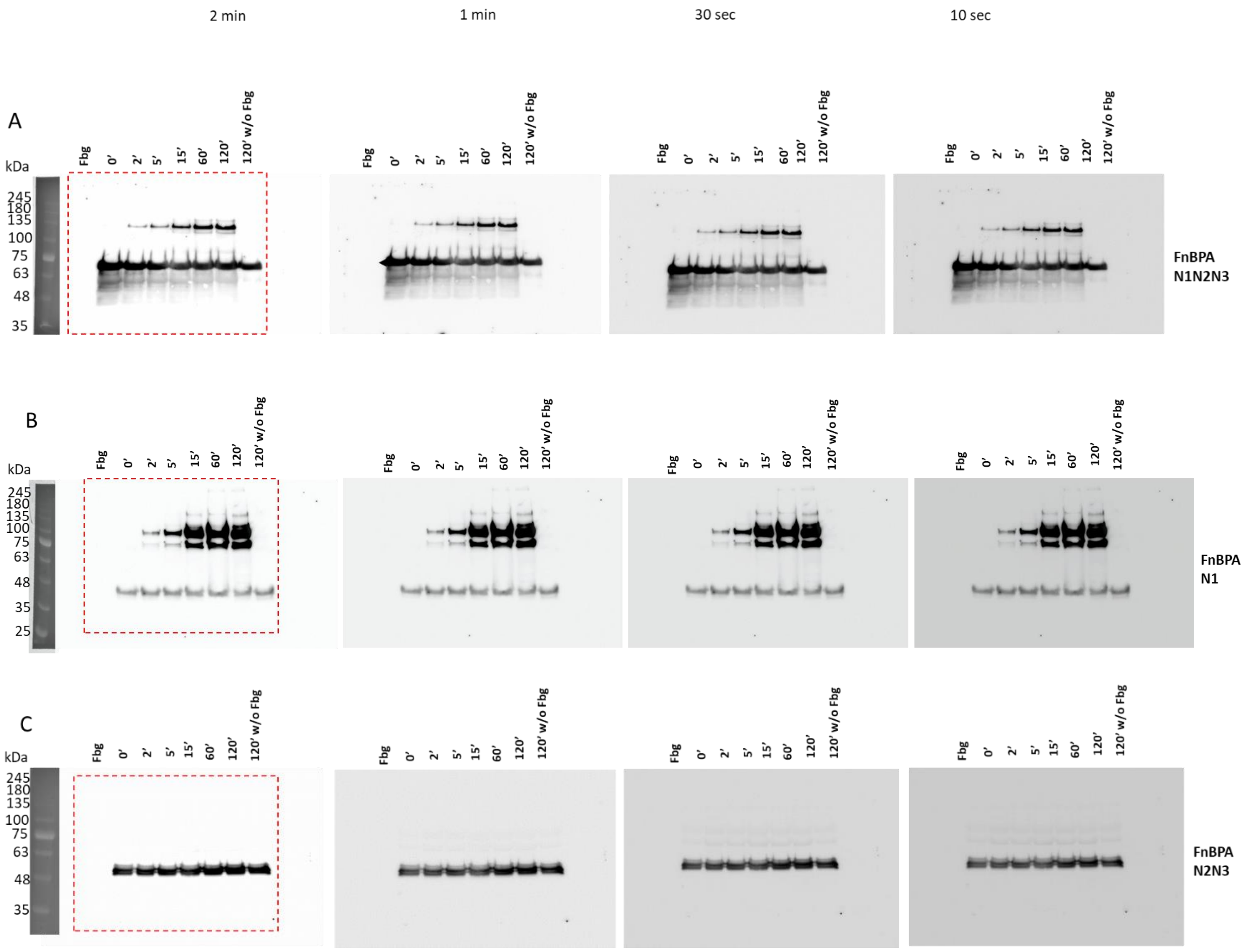

Supplementary Fig. 5. Full-length original Western blots shown in Fig. 6 of the main text are reported. The membranes were slightly cropped on the edges to make the results shown in Fig. 6 easier to understand. The area of each cropped membrane is indicated by a red dashed line. Multiple exposure times are reported on the top of the figure. The blots were not cut prior to hybridisation with antibodies. The standard protein size markers with the expected molecular weight are reported on the left. Incubation times on the top of each panel are also specified. Recombinant domains analysed are reported on the right of the panel (A, B and C).
